# Parental Awareness of Antibiotic Use for Upper Respiratory Tract Infection in Children in Jazan Region, Saudi Arabia

**DOI:** 10.7759/cureus.69438

**Published:** 2024-09-15

**Authors:** Ali A Al-Makramani, Ali M Shawish, Ahmed Y Sabi, Hafiz I Ghareeb, Faize A Faqih, Hassan A Daghas, Naif A Bahri, Abdulaziz Y Muyidi, Raghad H Bajawi, Elham A Maghrabi, Nesreen I Faqiri, Shahad M Hamithi, Shahad I Jawhari, Abdulrahman S Hamdi, Ebtihal E Eltyeb

**Affiliations:** 1 Department of Pediatrics, Jazan University, Jazan, SAU; 2 College of Medicine, Jazan University, Jazan, SAU; 3 College of Pharmacy, Jazan University, Jazan, SAU

**Keywords:** antibiotic misuse, antibiotic resistance, antibiotics, children, jazan, parental awareness, saudi arabia, upper respiratory tract infections

## Abstract

Background: Upper respiratory tract infections are a prevalent cause of morbidity in children, often managed in primary care settings. These infections are predominantly viral, but antibiotics are sometimes inappropriately prescribed. This misuse, driven by parental pressure and misconceptions, contributes to the growing problem of antibiotic resistance. The impact of parental awareness on antibiotic use practices is significant, as gaps in knowledge can lead to inappropriate use.

Methods: This study used a cross-sectional design to assess parents' awareness of antibiotic use for upper respiratory tract infections in children in Jazan, Saudi Arabia. Data were collected via a self-administered online questionnaire distributed through social media platforms. Participants were parents aged 18 years and older residing in Jazan. A snowball sampling technique was employed. The questionnaire assessed knowledge, attitudes, and practices regarding antibiotics.

Results: The study included 398 participants, with a nearly equal gender distribution of 198 females (49.7%) and 200 males (50.3%). Participants' ages were as follows: 148 (37.2%) were 18-30 years old, 162 (40.7%) were 31-40 years old, and 88 (22.1%) were over 40 years old. Regarding educational attainment, 29 (7.3%) mothers and 36 (9.0%) fathers had primary school education; 117 (29.4%) mothers and 128 (32.2%) fathers had secondary school education; 22 (5.5%) mothers and 33 (8.3%) fathers had intermediate school education; 209 (52.5%) mothers and 175 (44.0%) fathers held a bachelor’s degree; and 21 (5.3%) mothers and 26 (6.5%) fathers had a master’s degree. Most participants had a moderate income level (270, 67.8%) and resided in Bish (202, 50.8%). The main sources of information about antibiotics were doctors (242, 60.8%) and the internet (90, 22.6%). Perceptions varied, with 147 (36.9%) agreeing and 84 (21.1%) strongly agreeing that antibiotics can prevent complications from infections. A significant proportion believed that inappropriate use could lead to antibiotic resistance (172, 43.2% agreed; 129, 32.4% strongly agreed). Regarding the use of leftover antibiotics, 197 (49.5%) disagreed with using them without consulting a doctor. Interaction with pediatricians showed that 212 (53.3%) always followed pediatrician advice and instructions, while 66 (16.6%) rarely did.

Conclusion: The study highlights significant gaps in parental awareness regarding antibiotic use for upper respiratory tract infections in Jazan. Despite some understanding of antibiotic resistance, misconceptions about the efficacy and appropriate use of antibiotics persist. Addressing these gaps through targeted education and improving communication with healthcare providers could enhance antibiotic stewardship and reduce the risk of resistance.

## Introduction

Upper respiratory tract infections are a common cause of morbidity in children worldwide. These infections, often caused by viruses, are frequently managed in primary care settings. Despite the viral nature of most upper respiratory tract infections, antibiotics are sometimes inappropriately prescribed, driven by factors such as parental pressure, misconceptions about antibiotic efficacy, and the desire for quick symptom relief. Inappropriate use of antibiotics in such cases not only fails to address the underlying viral infection but also contributes to the growing problem of antibiotic resistance [1-3].

Antibiotic resistance is a critical public health issue that threatens the effectiveness of current antibiotic treatments. Resistance occurs when bacteria evolve mechanisms to withstand the effects of drugs that once killed or inhibited their growth. The overuse and misuse of antibiotics in both human and veterinary medicine have accelerated this process, leading to an increased prevalence of resistant infections [4-9]. This problem is exacerbated by gaps in public knowledge and understanding of appropriate antibiotic use, which can result in practices that further fuel resistance.

Significant research has focused on the impact of parental awareness and knowledge on antibiotic use practices. Studies have shown that parents’ understanding of when antibiotics are necessary, their beliefs about antibiotic efficacy, and their perceptions of potential side effects influence their decisions regarding their children’s treatment. In many cases, misinformation and lack of awareness contribute to the inappropriate use of antibiotics [8-11]. Therefore, assessing and improving parental knowledge is essential for promoting rational antibiotic use and mitigating the risk of resistance.

The Jazan region in Saudi Arabia presents a unique context for examining parental awareness of antibiotic use. With diverse socioeconomic backgrounds and varying levels of healthcare access, understanding local practices and perceptions regarding antibiotics can provide valuable insights. This study aims to assess the level of awareness among parents in Jazan about antibiotic use for upper respiratory tract infections in their children, identify gaps in knowledge, and inform strategies to improve antibiotic stewardship at the community level.

## Materials and methods

Study design

This study utilized a cross-sectional design to assess parents' awareness of antibiotic use for upper respiratory tract infections in children in Jazan, Saudi Arabia. A validated questionnaire was employed to gather data, which was distributed via social media platforms to reach a diverse sample of participants.

Study area and population

The research was conducted in the Jazan region, located in the southwestern part of Saudi Arabia. Participants included parents aged 18 years and older who resided in Jazan. Individuals without children, those unwilling to participate, and residents from outside Jazan were excluded from the study.

Sampling method

A snowball sampling technique was implemented. Social media platforms were utilized to disseminate the questionnaire, and participants were encouraged to share it within their networks. The sample size for this study was calculated using the Raosoft sample size calculator, and 377 participants were needed to reach a 95% confidence interval and a 5% margin of error.

Study questionnaire

The study employed a self-administered online questionnaire to gather data on parents' awareness of antibiotic use for upper respiratory tract infections in children. The questionnaire consisted of sections designed to assess participants' knowledge, attitudes, and practices regarding antibiotic use. It included multiple-choice questions and Likert scale items to evaluate the frequency and appropriateness of antibiotic use, as well as understanding of antibiotic resistance and potential side effects. The questionnaire was distributed through social media platforms to reach a broad audience and ensure diverse participation. Before distribution, the questionnaire was pre-tested to ensure the clarity and reliability of the questions.

Data collection and analysis

Data were collected using a validated, self-administered online questionnaire distributed through Google Forms and shared on social media platforms. Participants were encouraged to share the survey to maximize reach. The collected data were managed and analyzed using IBM SPSS Statistics for Windows, Version 27 (Released 2020; IBM Corp., Armonk, New York, United States). Descriptive statistics, including frequencies and percentages, were used to summarize categorical data, while continuous data were presented as means and standard deviations.

Ethical considerations

Approval for the study was obtained from the Research Ethics Committee at Jazan University. Informed consent was secured from all participants before their involvement in the study. The study adhered to ethical principles by ensuring participant anonymity, voluntary participation, and the right to withdraw without penalty. All data were stored securely and used solely for research purposes. The privacy and confidentiality of participants were strictly maintained throughout the study.

## Results

Demographic characteristics of study participants

The study included 398 participants with a nearly equal gender distribution of 198 females (49.7%) and 200 males (50.3%) (Table 1). Participants’ ages were distributed as follows: 148 (37.2%) were 18-30 years old, 162 (40.7%) were 31-40 years old, and 88 (22.1%) were over 40 years. For maternal education, 29 (7.3%) had primary school, 117 (29.4%) had secondary school, 22 (5.5%) had intermediate school, 209 (52.5%) held a bachelor’s degree, and 21 (5.3%) held a master’s degree. Paternal education levels included 36 (9.0%) with primary school, 128 (32.2%) with secondary school, 33 (8.3%) with intermediate school, 175 (44.0%) with a bachelor’s degree, and 26 (6.5%) with a master’s degree.

**Table 1:** Demographic characteristics of study participants (N=398) Data are represented in frequency and percentages; percentages may not sum to 100 due to rounding.

Characteristic		Frequency	Percent
Gender	Female	198	49.7%
	Male	200	50.3%
Age	18-30 years	148	37.2%
	31-40 years	162	40.7%
	Over 40 years	88	22.1%
Mother’s education level	Primary school	29	7.3%
	Secondary school	117	29.4%
	Intermediate school	22	5.5%
	Bachelor’s degree	209	52.5%
	Master’s degree	21	5.3%
Father’s education level	Primary school	36	9.0%
	Secondary school	128	32.2%
	Intermediate school	33	8.3%
	Bachelor’s degree	175	44.0%
	Master’s degree	26	6.5%

Socioeconomic characteristics and sources of information

Participants’ income levels indicated 18 (4.5%) were high, 270 (67.8%) were moderate, 82 (20.6%) were above average, 2 (0.5%) were very low, and 26 (6.5%) were low (Table 2). Most participants had 1-3 children under 14 years (295, 74.1%), while 71 (17.8%) had four children and 32 (8.0%) had more than four. Regarding pediatric visits, 296 (74.4%) visited within 1-2 days, 85 (21.4%) waited 3-4 days, and 17 (4.3%) waited more than four days. The main sources of information about antibiotics were doctors (242, 60.8%), the internet (90, 22.6%), friends (47, 11.8%), media (18, 4.5%), and radio (1, 0.3%).

**Table 2:** Socioeconomic characteristics and sources of information (N=398) Data are represented in frequency and percentages; percentages may not sum to 100 due to rounding.

Characteristic		Frequency	Percent
Income level	High	18	4.5%
	Moderate	270	67.8%
	Above average	82	20.6%
	Very low	2	0.5%
	Low	26	6.5%
Number of children under 14	1-3 children	295	74.1%
	4 children	71	17.8%
	More than 4 children	32	8.0%
Days before visiting a pediatrician	1-2 days	296	74.4%
	3-4 days	85	21.4%
	More than 4 days	17	4.3%
Sources of information on antibiotics	Friends	47	11.8%
	Media	18	4.5%
	Internet	90	22.6%
	Radio	1	0.3%
	Doctor	242	60.8%

Perceptions about antibiotics

Participants’ views on antibiotics varied (Table 3). The belief that antibiotics can prevent complications from infections was held by 147 (36.9%) who agreed and 84 (21.1%) who strongly agreed. In contrast, 15 (3.8%) strongly disagreed. Most participants agreed (172, 43.2%) or strongly agreed (129, 32.4%) that inappropriate use can lead to antibiotic resistance. Conversely, 130 (32.7%) strongly disagreed that antibiotics have no side effects, while only eight (2.0%) strongly agreed. The belief that antibiotics speed up recovery from flu symptoms was shared by 122 (30.7%) who agreed and 65 (16.3%) who strongly agreed, whereas 51 (12.8%) strongly disagreed. Regarding leftover antibiotics, 100 (25.1%) strongly disagreed and 197 (49.5%) disagreed with using them without a doctor’s consultation. A substantial number expressed a desire to change pediatricians if antibiotics were not prescribed in every visit (128, 32.2%) or not prescribed openly (122, 30.7%). Last, 86 (21.6%) felt there was excessive use of antibiotics in unnecessary cases, with 226 (56.8%) disagreeing.

**Table 3:** Antibiotic use for upper respiratory tract infections in children: perceptions about antibiotics (N=398) Data are represented in frequency and percentages; percentages may not sum to 100 due to rounding.

Statement	Strongly disagree	Disagree	Neutral	Agree	Strongly agree
Use of antibiotics can prevent complications from upper respiratory infections	30 (7.5%)	28 (7.0%)	109 (27.4%)	147 (36.9%)	84 (21.1%)
Inappropriate use of antibiotics can reduce their effectiveness and lead to antibiotic-resistant bacteria	15 (3.8%)	24 (6.0%)	58 (14.6%)	172 (43.2%)	129 (32.4%)
Antibiotics have no side effects	130 (32.7%)	134 (33.7%)	85 (21.4%)	41 (10.3%)	8 (2.0%)
Children with flu symptoms recover faster with antibiotics	51 (12.8%)	23 (5.8%)	137 (34.4%)	122 (30.7%)	65 (16.3%)
It's better to give antibiotics to any child with a high fever	106 (26.6%)	90 (22.6%)	116 (29.1%)	71 (17.8%)	15 (3.8%)
Most upper respiratory infections will improve without antibiotics	75 (18.8%)	13 (3.3%)	148 (37.2%)	110 (27.6%)	52 (13.1%)
Using leftover antibiotics at home for a child's symptoms without consulting a doctor	100 (25.1%)	197 (49.5%)	77 (19.3%)	19 (4.8%)	5 (1.3%)
Desire to change pediatricians due to not prescribing antibiotics in every visit	128 (32.2%)	112 (28.1%)	121 (30.4%)	33 (8.3%)	4 (1.0%)
Desire to change pediatricians due to not prescribing antibiotics openly	122 (30.7%)	129 (32.4%)	114 (28.6%)	27 (6.8%)	6 (1.5%)
Excessive use of antibiotics in unnecessary cases	86 (21.6%)	226 (56.8%)	63 (15.8%)	18 (4.5%)	5 (1.3%)

Sources and reasons for antibiotic use

Participants used various sources for antibiotic use (Table 4). Recommendations from relatives or colleagues were never considered by 69 (17.3%) and sometimes by 156 (39.2%). Pharmacist recommendations were never considered by 53 (13.3%) and sometimes by 124 (31.2%). Using old prescriptions was never done by 145 (36.4%) and sometimes by 100 (25.1%). The lack of time to visit a pediatrician was a reason for antibiotic use among 116 (29.1%) who never used antibiotics and 118 (29.6%) who sometimes did.

**Table 4:** Sources and reasons for antibiotic use in children (N=398) Data are represented in frequency and percentages; percentages may not sum to 100 due to rounding.

Source or reason	Never	Sometimes	Always	Often	Rarely
Recommendations from relatives or colleagues	69 (17.3%)	156 (39.2%)	28 (7.0%)	60 (15.1%)	85 (21.4%)
Pharmacist recommendations	53 (13.3%)	124 (31.2%)	81 (20.4%)	87 (21.9%)	53 (13.3%)
Using old or previous prescriptions	145 (36.4%)	100 (25.1%)	9 (2.3%)	39 (9.8%)	105 (26.4%)
No time to visit the pediatrician	116 (29.1%)	118 (29.6%)	8 (2.0%)	40 (10.1%)	116 (29.1%)

Interaction with pediatricians regarding antibiotics

Interactions with pediatricians about antibiotics varied (Table 5). Participants asked their pediatrician for antibiotics never 61 (15.3%) and sometimes 149 (37.4%). Requests via phone were made never by 154 (38.7%) and sometimes by 83 (20.9%). Discussions with pediatricians about antibiotics occurred never 64 (16.1%) and sometimes 106 (26.6%). Pediatricians informing participants about the diagnosis and need for antibiotics was reported as never done by 17 (4.3%) and sometimes done by 102 (25.6%). Following pediatrician advice and instructions were done always by 212 (53.3%), with 66 (16.6%) rarely following it.

**Table 5:** Interaction with pediatricians regarding antibiotics (N=398) Data are represented in frequency and percentages; percentages may not sum to 100 due to rounding.

Interaction or request	Never	Sometimes	Always	Often	Rarely
How often do you ask the pediatrician for antibiotics for your child?	61 (15.3%)	149 (37.4%)	52 (13.1%)	39 (9.8%)	97 (24.4%)
How often do you request antibiotics via phone from the pediatrician?	154 (38.7%)	83 (20.9%)	43 (10.8%)	19 (4.8%)	99 (24.9%)
How often do you discuss with the pediatrician whether antibiotics are necessary?	64 (16.1%)	106 (26.6%)	109 (27.4%)	50 (12.6%)	69 (17.3%)
How often does the pediatrician inform you about the diagnosis and need for antibiotics?	17 (4.3%)	102 (25.6%)	140 (35.2%)	83 (20.9%)	56 (14.1%)
How often do you follow the pediatrician’s advice and instructions?	7 (1.8%)	66 (16.6%)	212 (53.3%)	85 (21.4%)	28 (7.0%)

## Discussion

This study investigated parents' awareness of antibiotic use for upper respiratory tract infections in children within the Jazan region of Saudi Arabia. The findings highlight several key aspects of parental understanding and practices related to antibiotics, with implications for public health education and policy.

The results reveal that while a significant number of parents are aware of the potential benefits of antibiotics, there are considerable misconceptions. About 36.9% of participants believed that antibiotics could prevent complications from infections, which aligns with earlier studies indicating that many parents overestimate the efficacy of antibiotics for viral infections. The misconception that antibiotics can hasten recovery from the flu, held by 30.7% of participants, underscores the need for targeted educational interventions to correct this belief.

Most participants acknowledged that inappropriate antibiotic use could lead to resistance, with 75.6% agreeing or strongly agreeing with this statement. This is a positive finding, suggesting a baseline level of awareness about antibiotic resistance. However, the belief that antibiotics have no side effects was strongly disagreed with by a substantial portion of the sample (32.7%), indicating that a majority understands that antibiotics are not without risks. Despite this, there remains a significant proportion of parents who still use leftover antibiotics without consulting a healthcare provider, highlighting a gap in understanding regarding the safe use of antibiotics [10-14].

Interactions with pediatricians about antibiotics varied widely, with 53.3% of participants following their pediatrician's advice and instructions consistently. However, 16.1% of participants reported never discussing antibiotics with their pediatricians. This variability highlights the importance of fostering clear communication between parents and healthcare providers. Ensuring that pediatricians provide comprehensive information about the necessity and risks of antibiotics could improve adherence to medical advice and reduce inappropriate use [13-15].

The study underscores the necessity for enhanced educational programs targeting parents to address misconceptions about antibiotic use and resistance. Public health initiatives should focus on clarifying the role of antibiotics, addressing the risks of self-medication, and promoting appropriate consultation with healthcare providers. Additionally, integrating education about antibiotic stewardship into routine pediatric care could help bridge gaps in understanding and improve overall antibiotic practices.

Limitations

This study's reliance on a snowball sampling method and online distribution may limit the generalizability of the findings. Additionally, self-reported data may be subject to bias, as participants might provide socially desirable responses rather than accurate reflections of their practices.

## Conclusions

This study highlights significant gaps in parental awareness and practices concerning antibiotic use for upper respiratory tract infections in children within the Jazan region of Saudi Arabia. While many parents understand the risks of antibiotic resistance, misconceptions about the efficacy and appropriate use of antibiotics persist. The reliance on self-medication and variable interactions with pediatricians suggest that targeted educational initiatives are necessary to improve understanding and promote safer practices.

## References

[1] Al Harbi AA, Al-Ahmadi AF, Algamdi AG, Al-Dubai S (2023). Perception of antibiotic prescribing and resistance among hospital physicians in Medina City, Saudi Arabia. Cureus.

[2] Alajmi AM, Alamoudi AA, Halwani AA, Almangour TA, Almozain NH, Al-Jedai A, Tawfik EA (2023). Antimicrobial resistance awareness, antibiotics prescription errors and dispensing patterns by community pharmacists in Saudi Arabia. J Infect Public Health.

[3] Althagafi NS, Othman SS (2022). Knowledge, attitude, and practice of antibiotics use among primary healthcare physicians, Ministry of Health, Jeddah. J Family Med Prim Care.

[4] Alqahtani NS, Bilal MM, Al Margan AM, Albaghrah FA, Al Sharyan AM, Alyami AS (2024). Assessment of physicians' practice in implementing antibiotic stewardship program in Najran City, Saudi Arabia: a cross-sectional study. Pharmacy (Basel).

[5] Tan WL, Siti R, Shahfini I, Zuraidah A (2015). Knowledge, attitude and practice of antibiotics prescribing among medical officers of public health care facilities in the state of Kedah, Malaysia. Med J Malaysia.

[6] Darraj A, Almutairi M, Alhassan O (2023). Attitudes and practices of physicians toward law enforcement on dispensing antibiotics without prescription antibiotics: findings from a cross-sectional survey. J Family Med Prim Care.

[7] Alowfi A, Alghamdi R, Albogami D, Bukhari L, Khan MA, Almarhoumi L (2023). Assessment of knowledge, attitude, and practice of antibiotics prescription among healthcare residents at King Abdulaziz medical City, Jeddah, Saudi Arabia. Saudi Pharm J.

[8] Alothman A, Algwizani A, Alsulaiman M, Alalwan A, Binsalih S, Bosaeed M (2016). Knowledge and attitude of physicians toward prescribing antibiotics and the risk of resistance in two reference hospitals. Infect Dis (Auckl).

[9] Alrafiaah AS, Alqarny MH, Alkubedan HY, AlQueflie S, Omair A (2017). Are the Saudi parents aware of antibiotic role in upper respiratory tract infections in children?. J Infect Public Health.

[10] Alhomoud F, Almahasnah R, Alhomoud FK (2018). "You could lose when you misuse" - factors affecting over-the-counter sale of antibiotics in community pharmacies in Saudi Arabia: a qualitative study. BMC Health Serv Res.

[11] Alattas HA, Alyami SH (2017). Prescription of antibiotics for pulpal and periapical pathology among dentists in southern Saudi Arabia. J Glob Antimicrob Resist.

[12] El Zowalaty ME, Belkina T, Bahashwan SA (2016). Knowledge, awareness, and attitudes toward antibiotic use and antimicrobial resistance among Saudi population. Int J Clin Pharm.

[13] Baraka MA, Alboghdadly A, Alshawwa S (2021). Perspectives of healthcare professionals regarding factors associated with antimicrobial resistance (AMR) and their consequences: a cross sectional study in Eastern Province of Saudi Arabia. Antibiotics (Basel).

[14] Baadani AM, Baig K, Alfahad WA, Aldalbahi S, Omrani AS (2015). Physicians' knowledge, perceptions, and attitudes toward antimicrobial prescribing in Riyadh, Saudi Arabia. Saudi Med J.

[15] Hajjar W, Alnassar S, Al-Khelb S, Al-Mutairi S, Al-Refayi N, Meo SA (2017). Antibiotics use and misuse in upper respiratory tract infection patients: knowledge, attitude and practice analysis in University Hospital, Saudi Arabia. J Pak Med Assoc.

